# Establishment of Carotid Artery Dissection and MRI Findings in a Swine Model

**DOI:** 10.3389/fneur.2021.669276

**Published:** 2021-06-16

**Authors:** Jing Peng, Min Wu, Desislava Met Doycheva, Yi He, Qiongzhen Huang, Wei Chen, Nathanael Matei, Jun Ding, Kangning Chen, Ningbo Xu, Zhenhua Zhou

**Affiliations:** ^1^Department of Neurology, Southwest Hospital, Third Military Medical University, Chongqing, China; ^2^Departments of Physiology and Pharmacology, Basic Sciences, School of Medicine, Loma Linda University, Loma Linda, CA, United States; ^3^Department of Microsurgery, Chongqing Hengsheng Surgical Hospital, Chongqing, China; ^4^Department of Interventional Therapy, Zhujiang Hospital, Southern Medical University, Guangzhou, China; ^5^Department of Radiology, Southwest Hospital, Third Military Medical University, Chongqing, China; ^6^Department of Ultrasound, Southwest Hospital, Third Military Medical University, Chongqing, China

**Keywords:** dissection, carotid artery, ischemic stroke, animal model, swine

## Abstract

Carotid artery dissection (CAD) is the leading cause of ischemic stroke in young patients; however, the etiology and pathophysiology of CAD remain largely unknown. In our study, two types of dissections (length × width: 1.5 cm × 1/3 circumference of intima, Group I, *n* = 6; or 1.5 cm × 2/3 circumference of intima, Group II, *n* = 6) were created between the media and intima. Ultrasound (within 2 h after dissection) showed a dissociated intima in the lumen and obstructed blood flow in the surgical area. Digital subtraction angiography (DSA, 72 h after dissection), magnetic resonance imaging (MRI, 72 h after dissection), and hematoxylin–eosin (H&E, 7 days after dissection) staining confirmed stenosis (33.67 ± 5.66%) in Group I and total occlusion in Group II. In 10 out of 12 swine, the CAD model was established using a detacher and balloon dilation, and morphological outcomes (stenosis or occlusion) after CAD were determined by the size of intimal incision.

## Introduction

Cervical artery dissection (CeAD) accounts for 1–2% of all ischemic strokes and accounts for 10%−25% of youth ischemic strokes (<45 years of age) without traditional vascular risk factors (1–3). Depending on the affected vessel, CeAD is divided into carotid artery dissection (CAD) or vertebral artery dissection (VAD) (4). CAD accounts for majority of CeAD, which usually occurs spontaneously or secondary to trauma (5). Cerebral ischemia related to dissection is thought to be usually embolic, from the intra-luminal thrombi forming at the site of the intimal tear (6, 7). Regular treatments after artery dissection include anticoagulation, antiplatelet therapy, angioplasty with or without stenting, or conservative observation without specific medical therapy (5, 8, 9). Due to the dearth of successful animal models, there remains a paucity of information on the pathogenesis of CeAD. Moreover, the prophylactic strategy for CAD patients, who are at risk for ischemic stroke, is largely guided by empirical studies (10–12). Therefore, the purpose of this study is to produce a suitable animal model of CAD using the common carotid artery. Morphological changes of CAD in acute or subacute periods were assessed by ultrasound, digital subtraction angiography (DSA), magnetic resonance imaging (MRI), and hematoxylin–eosin (H&E) staining. Establishing a successful animal model will aid future studies focused on the development of prognostic information and suitable treatments for CeAD.

## Materials and Methods

### Animals

Twelve male Bama mini pigs (Guilin, China), with an average weight of 30–35 kg, were provided by the Laboratory Animal Center of the Third Military Medical University. The study was performed using a protocol approved by the Institutional Animal Care and Use Committee of the Third Military Medical University.

### Methods

The swine were anesthetized with an intramuscular or subcutaneous injection of ketamine hydrochloride (33 mg/kg) (Sigma-Aldrich, St. Louis, MO, USA). Once the animal was immobilized by anesthesia, it was transferred to the preparation area. Then, surgical sites were shaved, and the swine was moved from the preparation area to the surgical site. After tracheal intubation, 1–2% isoflurane and 0.5–1.5 L/min oxygen were given by a mechanically ventilated closed-loop anesthesia machine. All the surgical procedures were performed under sterile conditions with the aid of a surgical microscope. Unfractionated heparin (UFH, 100 IU/kg) (Sigma-Aldrich, St. Louis, MO, USA) was administrated intravenously for systemic anticoagulation.

**Figure 1:**
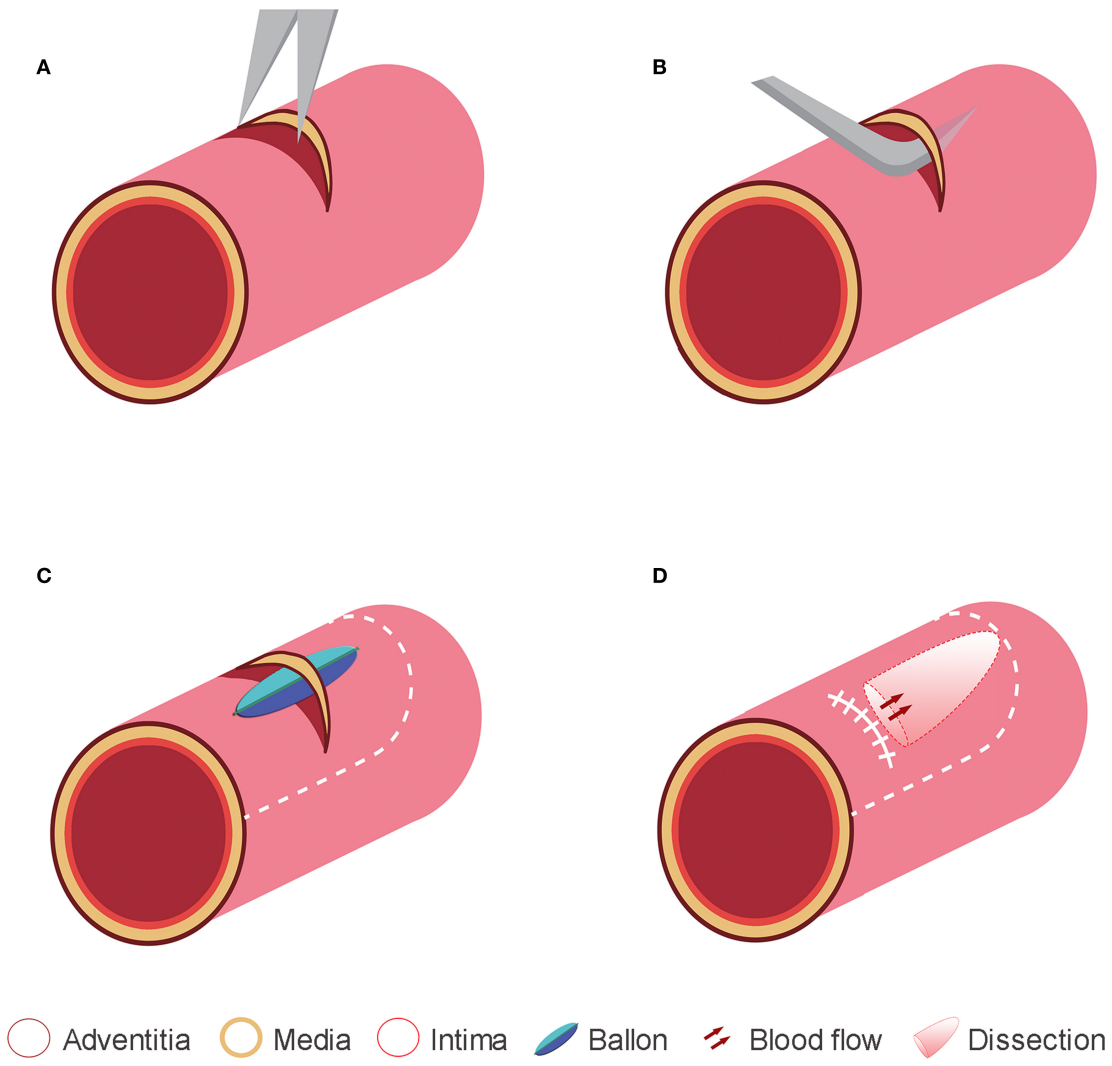
Representative diagrams to illustrate the method used for creating the experimental dissections. **(A)** In a swine common carotid artery, a small incision was made in the adventitia and media layers under the microscope. **(B)** The intima was dissected from the media by using a detacher that was passed through the adventitia and media incisions. **(C)** The intima was also dissected from the media using balloon dilation. **(D)** The adventitia and media incisions were tightly closed, and a dissection plane was made between the intima and media layer.

With the swine fixed in the supine position, a 10-cm longitudinal paramedian skin incision was made in the right anterior aspect of the neck, adjacent to the sternocleidomastoid muscle, and then the right common carotid artery (RCCA) was isolated. After vascular clamps were placed distally and proximally, approximately one-third or two-third the circumference of the adventitia and media was cut transversely, limited to the adventitia and media layers (Figure 1A). The media was then dissected from the intima with the help of detachers (Figure 1B). After a 0.5-cm length × 0.5-mm width dissection was made between the two layers, a wrapped balloon was inserted into the space between the media and intima (Figure 1C). Subsequently, detachers and balloon dilation were alternatingly used to mechanically separate the cavity to 1.5-cm length × 1/3 circumference (Group I, *n* = 6) or 1.5-cm length × 2/3 circumference (Group II, *n* = 6) of intima width (Figure 1C). Then, a continuous eversion suture (8-0 Prolene) was used to close the adventitia and part of the media (Figure 1D). After checking that the vessel was filled and no oozing resulted from the incised segment of the adventitia, the muscle, adipose, and skin were successively closed. In addition, the swine were started on an anti-infection (cefazolin sodium, 1 g) (Sigma-Aldrich, St. Louis, MO, USA) treatment *via* intramuscular injection every 12 h for a total of 7 days. No antiplatelet or anticoagulation medication was administered before, during, or after dissections.

Immediately, following these procedures, external changes in the dissected portion were observed macroscopically. Then, carotid artery stenosis screening was assessed within 2 h post dissection by ultrasound (Philips IU22, Philips Medical Systems, Holland) concurrent with the evaluation of both the macroscopic appearance of dissection and/or of hematoma and the flow characteristics in the common carotid artery.

DSA and MRI were performed to evaluate subacute changes at 72 h post-surgery. Briefly, after general anesthesia, the right groin was prepped and draped in a sterile fashion. A 5F arterial sheath was inserted from the right femoral artery, and a 5F guiding catheter was guided to the origin of RCCA using a guide wire under X-ray fluoroscopy. Once the catheter was in place, the dye was sent through the catheter. X-ray images were taken to observe the dye movement through the dissected artery. MRI data were collected in all cases by a 3.0-T MRI scanner (Siemens, Germany) after DSA.

At 7 days post dissection, the dissected arteries or contralateral common carotid arteries were harvested and fixed in 10% formaldehyde for tissue histology. They were then stained with H&E and examined using light microscopy.

## Results

Of the 12 animals, 11 survived the surgery. One animal died from anesthesia during the surgery in Group I, and one animal was excluded due to a rupture of the distal intima of the common carotid artery in Group II. Ten unilateral CADs were successfully observed after recanalization.

**Figure 2:**
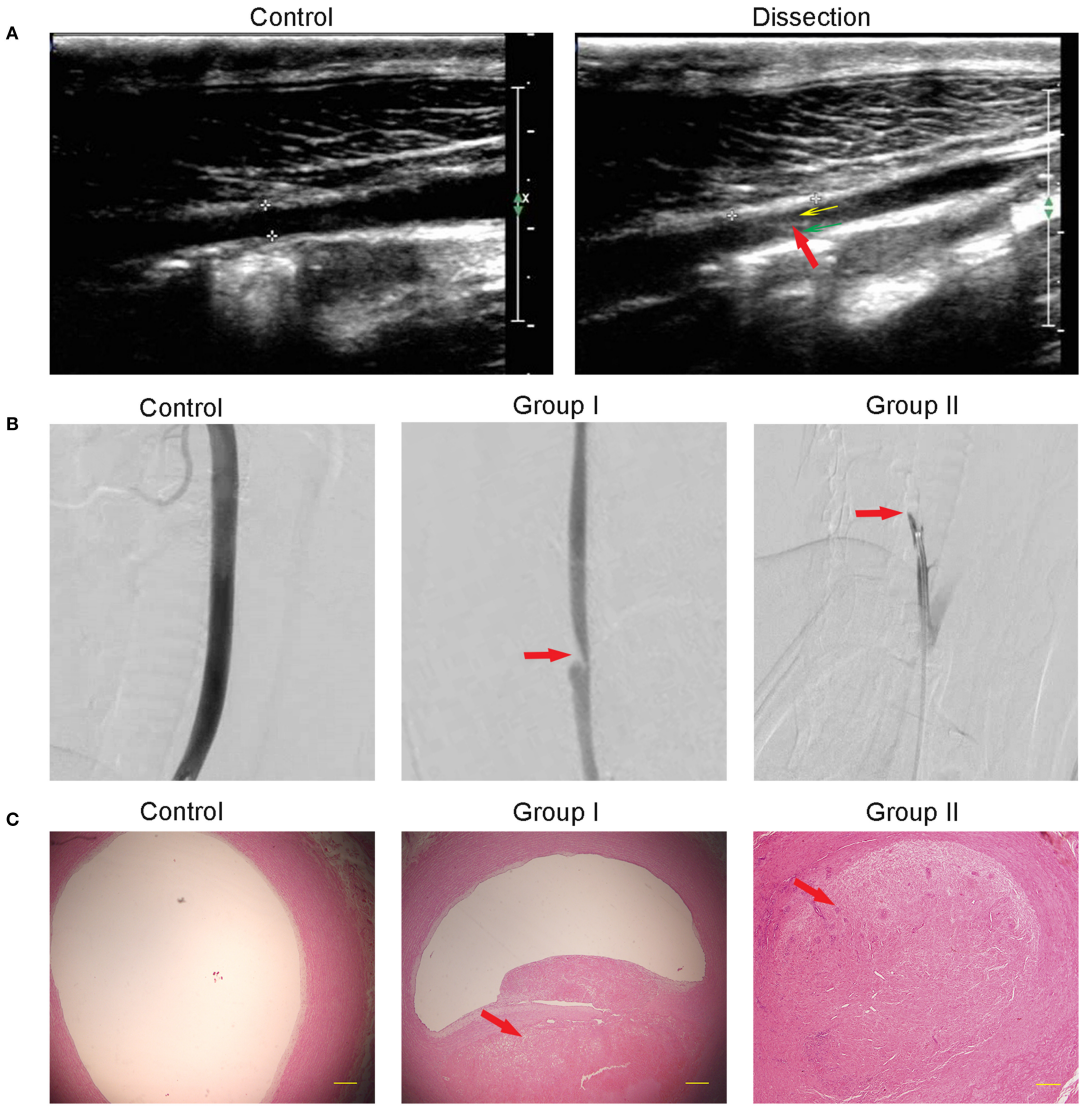
Morphological changes after dissection. **(A)** Ultrasound showed a normal artery in the control and the false lumen (yellow arrow), true lumen (green arrow), and dissociated intima (red arrow) in the dissection. **(B)** DSA showed that the intramural hematoma formation caused arterial lumen stenosis (Group I) or occlusion (Group II). The red arrow points to the dissection location. **(C)** H&E staining showed that the subintima hematoma, which is partly organized, caused stenosis in Group I or full occlusion in Group II. Contralateral arterial lumen showed no stenosis or occlusion.

Immediately following recanalization, all lesions showed an abrupt formation of subintimal hematoma due to the influx of blood through the intimal entry zone. Ultrasound monitoring showed the intima floating in true and false artery lumens in 10 swine while no double lumens were observed in contralateral arteries (Figure 2A). The DSA demonstrated stenotic changes in the artery in all 10 swine except in contralateral arteries. Stenosis was marked at the site of the intimal entry zone, and the dissection occurred in the lesion near the origin of the RCCA in Group I, with an average stenosis of 33.67 ± 5.66%. No stenosis extended over a long segment, representing total occlusion in all Group II cases (Figure 2B). H&E staining results showed that, on the transverse cross section, the dissection cavity was microscopically presented as stenosis (Group I) or occlusion (Group II) due to thrombus formation between the intima and media (Figure 2C).

**Figure 3:**
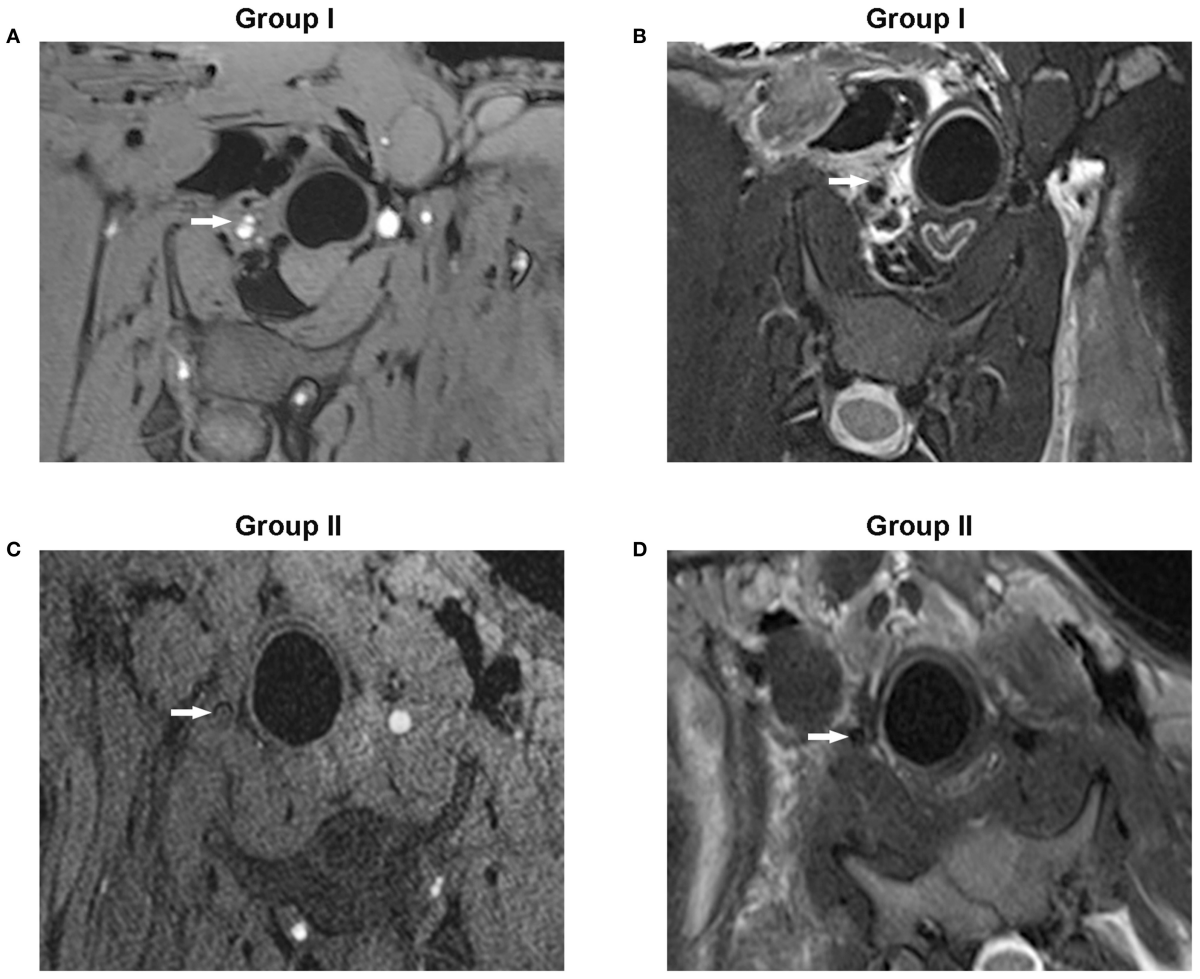
MRI conducted with T1-weighted gradient echo or T2-weighted imaging techniques showed the coronal slices of the dissection of the RCCA. **(A)** T1-weighted gradient echo images showed hyperintense true and false lumens while the dissociated intima emitted a low signal (Group I). **(B)** T2-weighted images showed a true lumen with a low signal, but both the false lumen and dissociated intima displayed a slightly higher signal (Group I). **(C)** No high signal of circulating blood flow was observed in the arterial lumen in T1-weighted gradient echo images (Group II). **(D)** T2-weighted images showed no low signal of circulating blood flow (Group II). The white arrow points to the arterial lumen.

MRI has been used to detect intramural hematoma abnormalities through T1-weighted gradient echo (T1) and T2-weighted imaging techniques (T2). In Group I, T1 demonstrated a double lumen in RCCA; both true and false lumens presented an abnormal signal, while the intima between them showed a low signal intensity (Figure 3A). T2 showed the double-lumen phenomenon, a true lumen (low signal), false lumen, and intima (slightly higher signal) (Figure 3B). In Group II, T1 demonstrated a low signal of the tube with no high signal of blood flow, indicating an occlusion of the RCCA (Figure 3C). Consistently, the low-signal circulating blood flow was not observed in the T2 images (Figure 3D).

## Discussion

CeAD is increasingly commonly identified as the cause of cerebrovascular accidents, which are defined by the presence of an intramural hematoma in a cervical artery (13). CeAD may affect the internal carotid and/or the vertebral arteries in their various extracranial segments while the former is the most commonly affected vessel (2, 14). Traumas and primary disease of the arterial wall are the main predisposing factors (15), but the etiology and pathogenesis of CeAD remain unknown in the majority of cases (16).

Due to the lack of successful CeAD models, a lot of uncertainty and/or controversy remains with respect to treatment strategies. Hence, there remains an urgent need to develop a model that closely resembles CeAD in patients. Kahler et al. created a sub-adventitia intra-medial dissection plane in the arterial wall of the internal carotid artery in a New Zealand White rabbit model (17). However, arterial thrombus or narrowing was not observed at the second stage of the operation, which was inadequate for a correlation to human CeAD. Subsequently, Takeshi Okamoto et al. made an elliptical defect or longitudinal incision in the intima and media layer which caused a perforation of both layers resulting in aneurysm or stenosis in the common carotid arteries in a mongrel dog model (18). Based on this study, the size of the intimal entry zone determines the morphological changes observed after experimental CAD. However, in the clinical setting, majority of patients exhibit a subintimal dissection without perforation of the media layer. In the present study, we performed microsurgery to separate the intima and media. With the help of balloon dilation, we further expanded the space between the two layers and tightly sutured the media and adventitia which helped to keep the media layer intact.

Mini pigs are widely used as biomedical models (19). Their cervical artery anatomy, size, structure, and distribution are comparable to human vasculature (20, 21). For example, the swine common carotid artery has a diameter of 4 to 5 mm, closely resembling the common carotid artery diameter of humans (22). Taken together, the swine dissection model can simulate CAD in humans.

Given the anatomical similarities between humans and swine, many of the same clinical imaging techniques can also be used in the swine model (23). Ultrasound depicted the formation of a true lumen and a false lumen and the presence of a dissociated intimal flap in both groups. DSA demonstrated irregular stenosis (Group I) or full occlusion (Group II) of the artery. MRI enabled visualization of the hyperintense mural hematoma which appeared with a “crescent” shape (Group I) or vessel occlusion (Group II). H&E staining showed lumen stenosis (Group I) or vessel occlusion (Group II). Taken together, stenosis or occlusion was determined by the size of the intimal incision, and all the pathological results confirmed the presence of dissection and thrombosis in the surgical area, confirming a successful representation model of human CAD.

However, this model has several limitations. First, in our model, there was no evidence of progression of the dissection beyond the initial surgery. In contrast, progression of the dissection and CAD manifestations such as dissection leading to aneurysm and intimal dissection flap were observed in clinical settings (24, 25). This may be explained in part by the absence of mural pathology due to the use of healthy swine with no preexisting vascular pathologies (17). Second, in the subintimal dissections, the hematoma compressed the arterial lumen, leading to a variable degree of stenosis or even occlusion (14, 25). In human subjects, thrombus formation may occur, antegrade to the site of dissection, and may result in emboli passing into the cerebral circulation, which is thought to be the major driving force of ischemic stroke pathogenesis (26, 27). However, in our model, we did not detect embolism in the brain, but we did observe morphological changes in the surgery site. Additional studies are needed to investigate cerebral perfusion and thrombus formation in the swine brain (26). Finally, our model was created by a traumatic dissection, and other models such as spontaneous dissection may be developed through biochemical or molecular methods.

In conclusion, we established a CAD model in swine. This model can be easily adapted to suit individual study designs and investigate a variety of possible interventions. This model will be a useful tool for translational research into the pathophysiology of CAD and is an ideal testing platform for novel biological approaches targeting regenerative medicine (28, 29).

## Data Availability Statement

The original contributions presented in the study are included in the article/supplementary material, further inquiries can be directed to the corresponding author/s.

## Ethics Statement

The animal study was reviewed and approved by Institutional Animal Care and Use Committee of Third Military Medical University.

## Author Contributions

Conceptualizing and designing were performed by JP, MW, YH, WC, JD, KC, and ZZ. Drafting the article was done by NX, JP, MW, DD, QH, and NM. Approving the final version of the manuscript on behalf of all authors was done by ZZ. Critically revising the article and reviewing the submitted version of the manuscript was done by all authors.

## Conflict of Interest

The authors declare that the research was conducted in the absence of any commercial or financial relationships that could be construed as a potential conflict of interest.
